# Evaluation of the autonomic nervous system in autochthonous patients from Amazon with acute Chagas disease treated with benznidazole

**DOI:** 10.1371/journal.pntd.0014022

**Published:** 2026-03-12

**Authors:** Mônica Regina Hosannah da Silva e Silva, Débora Raysa Teixeira de Sousa, Jessica Vanina Ortiz, Matheus Martins Monteiro, Alba Regina Jorge Brandão, Marcia Regina Silva e Silva, Susan Smith-Doria, Melissa de Sousa Melo Cavalcante, Katia do Nascimento Couceiro, Guilherme Peixoto Tinoco Arêas, Jorge Augusto de Oliveira Guerra, Maria das Graças Vale Barbosa Guerra, João Marcos Bemfica Barbosa Ferreira

**Affiliations:** 1 Programa de Pos-graduação em Medicina Tropical, Universidade do Estado do Amazonas, Manaus, Amazonas, Brazil; 2 Fundação de Medicina Tropical Doutor Heitor Vieira Dourado, Manaus, Amazonas, Brazil; 3 Real e Benemérita Associação Portuguesa de Beneficência, São Paulo, São Paulo, Brazil; 4 Escola Superior de Ciências da Saúde, Universidade do Estado do Amazonas, Manaus, Amazonas, Brazil; 5 Universidade Federal do Amazonas, Manaus, Amazonas, Brazil; Universidade Federal de Minas Gerais, BRAZIL

## Abstract

Chagas disease, a neglected tropical disease, is endemic in the Amazon region, where oral transmission predominates. Autonomic nervous system (ANS) impairment is a recognized pathophysiological mechanism contributing to disease progression, including Chagas cardiomyopathy. This study aimed to assess ANS function in patients with acute Chagas disease from the Amazon, evaluating responses pre- and post-benznidazole treatment. We included 28 acute-phase patients and 20 healthy controls. Participants underwent comprehensive cardiac evaluations, including 12-lead ECG, echocardiogram, 24-hour Holter monitoring, treadmill stress testing, and 5-minute heart rate variability (HRV) assessment. HRV was analyzed across time, frequency, and nonlinear domains, with statistical comparisons performed between groups and within the patient cohort. The study population predominantly comprised individuals from rural Amazonian municipalities (89.3%), with oral transmission accounting for 85.7% of infections. While resting ECGs were normal in 60.7%, diffuse ventricular repolarization was the most common abnormality (21.4%). Before treatment, 24-hour HRV showed significant reductions in SDANN and SDNN, indicating sympathovagal imbalance. For 5-minute HRV, significant alterations were observed across time (rMSSD, SDNN), frequency (LF, HF, LF/HF ratio), and nonlinear domains, reflecting reduced parasympathetic tone. Intragroup comparisons (pre- vs. post-treatment) further reinforced the sustained sympathovagal imbalance and parasympathetic inhibition. These findings highlight persistent autonomic modulation alterations, characterized by sympathovagal imbalance and reduced parasympathetic activity, in acute Chagas disease patients from the Amazon. Such dysfunction may predispose individuals to long-term structural cardiac changes and arrhythmias, underscoring the critical need for continued monitoring and potential targeted interventions to address autonomic imbalance in this vulnerable population.

## Author summary

Chagas disease is a neglected tropical disease, globally prevalent, with the potential for progression to severe forms. It is endemic to the Amazon region, presenting in both acute and chronic forms, with oral transmission being one of the most frequent modes of Chagas disease acquisition in this area. The primary pathogenic mechanisms proposed to explain disease progression include direct tissue damage, an immune response against the parasite with the potential for autoimmunity, and autonomic nervous system dysfunction. The aim of this study was to evaluate the autonomic nervous system response in patients diagnosed with acute Chagas disease, residing in the Amazon region and receiving care at the outpatient clinic of the Fundação de Medicina Tropical Dr. Heitor Vieira Dourado.

## Introduction

Chagas disease (CD), also known as American Trypanosomiasis, is an endemic, transmissible, and potentially fatal anthropozoonosis caused by the parasite *Trypanosoma cruzi*. It is classified by the World Health Organization (WHO) as one of the neglected tropical diseases. CD has a wide distribution across the Americas, being observed from the southern United States to Patagonia [1,2]. It was discovered by Carlos Ribeiro Justiniano Chagas in 1909, who identified the etiological agent, the form of transmission, the natural history of the disease, and its correlation with the chronic inflammatory process of the myocardium [3,4].

The transmission of Chagas disease occurs through vectorial, oral, transplacental routes, organ and tissue transplantation, accidents involving biological materials, and the sharing of needles/syringes contaminated by illicit drug users. The migratory flow of Latin Americans has been pointed out as a form of dissemination in non-endemic countries [2–8].

The Amazon region, long considered by many as a low-risk area, has recently been the scenario for numerous acute and chronic cases of the disease. The *T. cruzi* transmission cycle in this region does not involve domiciliated vectors, being sustained by an enzootic cycle with wild vectors implicated in cases associated with oral or extradomiciliary vectorial transmission [2,9–23].

The physiopathogenesis of Chagas disease is multifactorial, comprising two phases: acute and chronic. The acute phase occurs due to primary infection or reactivation of a chronic phase and is related to intense tissue parasitism. In this phase, some individuals infected by *T. cruzi* may develop diffuse myocarditis, characterized by low and persistent inflammatory intensity [2]. The main proposed pathogenic hypotheses to explain the onset and progression of Chronic Chagas Cardiomyopathy (CCC) are: 1) direct parasite-induced tissue damage; 2) indirect inflammatory/immunological tissue damage; 3) neurogenic disorders; and 4) microvascular disorders [10,24–27].

Neurogenic disorders are characterized by autonomic denervation. In CD, this denervation may be implicated as one of the causes for alterations in reflex heart rate control in patients with CCC. These alterations lead to the deprivation of the tonic inhibitory action of the parasympathetic system on the sinus node and the absence of the vagus-mediated mechanism for chronotropic control in response to transient changes in blood pressure or venous return [14,15,28–38]. These autonomic nervous system alterations can be detected before the development of ventricular dysfunction in indeterminate forms and early stages of the chronic phase (e.g., indeterminate form of Chagas disease, digestive form) of CD. Evidence suggests a disorganized, random, and incomplete restoration of these parasympathetic neuroeffector junctions, postulating the possibility of a parasympathetically denervated cardiomyopathy where antagonism of catecholaminergic effects would not occur [29,30]. In CD, it is suggested that the depression of the cardiac parasympathetic system contributes to exacerbating inflammation during the chronic phase [39,40].

One of the mechanisms for evaluating the autonomic nervous system is the analysis of heart rate variability (HRV). Several studies have demonstrated autonomic alterations in different stages and forms of CD, both in short (5-minute) and long (24-hour) periods. Heart rate variability (HRV) analysis has been used to assess ANS function for both the detection of autonomic dysfunction and the evaluation of sympathovagal modulation. It is a non-invasive, feasible measure with the capacity to identify phenomena related to the autonomic nervous system in healthy individuals, athletes, and patients [2,15,41–44]. The exercise stress test can be utilized as a tool for investigating the autonomic nervous system by evaluating heart rate responses to exertion [36,45].

Chronic Chagas Cardiomyopathy is considered a serious public health problem in endemic areas of Latin America, representing one of the leading causes of heart failure and sudden death. In the Amazon region, Chagas disease appears to have lower morbidity compared to other endemic areas, attributed either to low parasitemia or reduced parasite pathogenicity, or both, in this region. Therefore, there is a need to acquire more knowledge about the natural history of the disease in the Amazon region to enhance the clinical approach for patients with CD and to enable the development of surveillance strategies substantiated by new observations.

In the Amazon, ANS involvement has not yet been studied. Therefore, this study proposed to evaluate the autonomic nervous system function in patients diagnosed with acute Chagas disease, autochthonous to the Amazon, attended at the outpatient clinic of the Tropical Medicine Foundation Heitor Vieira Dourado, during the acute phase, pre- and post-benznidazole treatment.

## Materials and methods

### Ethics statement

Institutional Review Board Statement: This study is part of a larger project titled “Evaluation of cardiac function, autonomic nervous system, and metabolic and inflammatory activity in the various evolutionary forms of Chagas Disease in autochthonous Amazonian patients,” approved under call for proposals number 30/2013 from the Amazonas State University and FAPEAM, with funding for execution, and by the Research Ethics Committee of the Fundação de Medicina Tropical Doutor Heitor Vieira Dourado, CAEE number 31812914.9.0000.5616, Approval number 923.701 – May, 2016. Informed consent was obtained from all subjects involved in the study, in agreement with Resolution 466/12 of the Brazilian National Health Council and ethical guidelines of the 1975 Declaration of Helsinki.

### Study population

This was a longitudinal, case-control study, with an inclusion period from January 2019 to July 2022. The study population comprised individuals diagnosed with Acute Chagas Disease (ACD) treated at the Fundação de Medicina Tropical Heitor Vieira Dourado.

The diagnosis of Acute Chagas Disease (ACD) was established based on a comprehensive set of criteria. These included a strong clinical suspicion, derived from suggestive signs and symptoms of the disease, coupled with the presence of compatible epidemiological determinants and conditioning factors, such as documented outbreaks among family members or close contacts. A crucial component of the diagnostic process was the etiological confirmation, which involved identifying parasite evidence in circulating blood through either thick or thin blood smear examination. Furthermore, patients diagnosed specifically through the Amazon Malaria Surveillance System were classified as being in the acute phase of the disease [2,46].

These patients were divided into 3 groups: a. Group G1 – pre-treatment, acute-phase patients presenting within 48–72 hours of symptom onset; b. Group G2 – follow-up group, consisting of patients post-benznidazole treatment (between 6 and 12 months); c. Group G3 – healthy control group.

All patients received medical follow-up at FMT – HVD and were invited to participate in the research. To formalize their participation in the study, these invited individuals signed the Informed Consent Form (ICF). In case of refusal to participate, they would continue to receive all appropriate medical support and treatment.

Groups G1 and G2 were composed of patients and designated as the case group. The control group was composed of healthy individuals with negative serology for CD, selected from blood donors in Manaus city. Patients were invited to participate in the research and were informed about all procedures performed and the intended use of the information collected from the examinations. The groups were subjected to clinical examination and assessed according to inclusion and exclusion criteria.

The inclusion criteria were patients with a confirmed diagnosis of Acute Chagas Disease, autochthonous to the Amazon region, aged between 18 and 60 years; both genders, with parasitological diagnosis treated with benznidazole for 2 months. Individuals from other regions of Brazil were not included. Patients with moderate to severe valvular heart disease, individuals with thyroid diseases, chronic obstructive pulmonary disease, diabetes mellitus, carriers of cardiac arrhythmias such as: atrial fibrillation, advanced atrioventricular block; pacemaker bearers, and individuals with heart failure, functional class III or IV (NYHA) were excluded.

### Procedures

After outpatient care at FMT - HVD, patients with acute disease were clinically evaluated and underwent blood collection for laboratory assessment. These individuals received benznidazole treatment for 2 months. Patients underwent cardiological examinations such as electrocardiogram, 5-minute heart rate variability assessment, transthoracic echocardiogram, and 24-hour dynamic electrocardiography (Holter).

The cardiological examinations were performed as follows: electrocardiogram and heart rate analysis at FMT-HVD, transthoracic echocardiogram at Fundação Hospital Universitário Francisca Mendes or UltraCor Clínica Cardiológica, and dynamic electrocardiography at Clínica de Arritmias de Manaus. During the initial clinical-cardiological evaluation, the patient was classified according to their functional class by NYHA.

#### Electrocardiogram.

The 12-lead electrocardiogram (ECG) was performed in rest and all examination reports adhered strictly to the 2022 guidelines established by Brazilian Society of cardiology (SBC) [47].

#### Transthoracic echocardiogram.

Echocardiography, a non-invasive imaging technique, provides crucial anatomical, functional, and hemodynamic insights widely utilized in Cardiology. A primary application is the assessment of left ventricular (LV) systolic function, a key prognostic indicator across various cardiovascular conditions, including Chronic Chagas Cardiomyopathy (CCC) [48]. For this study, two-dimensional transthoracic echocardiograms were conducted using GE VIVID – 5S or VIVID - 7 machines. Standard cardiac chamber measurements, encompassing left ventricular systolic and diastolic diameters (LVSd, LVDd), right ventricular diastolic diameter (RVDd), and indexed left atrial volume, were acquired, alongside comprehensive evaluations of ventricular systolic and diastolic functions.

#### Heart rate variability assessment.

Autonomic Nervous System (ANS) function was assessed through Heart Rate Variability (HRV) evaluation over a short period (5 minutes) and via dynamic electrocardiography. For the 5-minute heart rate assessment, a POLAR heart rate monitor, model V800, was used. Volunteers were positioned in supine decubitus, in a calm environment with controlled temperature (22–24 degrees Celsius), without visual or auditory stimuli, with prior instruction to maintain this position throughout heart rate acquisition. To minimize external influence on the ANS response, the volunteer was instructed to remain as still as possible. Second-by-second recording of heartbeats began for a total period of 5 minutes.

For impulse acquisition, a strap with electrodes was placed at the xiphoid process, at the end of five minutes, the recording was stopped. The choice to record the time series of ECG RR intervals using this device was made because it is an instrument previously validated as an accepted alternative for this purpose, as well as for its ease of operation [44,49–51]. HRV analysis was performed using Kubios 2.0 analysis software for Windows (Biomedical Signal and Medical Imaging Analysis Group, Department of Applied Physics, University of Kuopio, Finland).

Dynamic electrocardiography, or Holter monitoring, is a non-invasive examination where the patient wears a device that records heart rhythm for 24 hours. Through software, these recordings are processed, and heart rate variability is calculated. Tracings with a minimum of 85% sinus heartbeats were evaluated. Using a patient-activated marking device, the user can correlate arrhythmic phenomena with time during the examination, facilitating the localization of the event, symptom, or activity at the time of analysis. Every Holter examination should allow for the reproduction of the 24-hour recordings in compact tracings and conventional scale, in addition to presenting data in the form of tables, charts, and histograms. A DMS brand recorder and compatible reading software were used [42,43,52].

Patients in the post-treatment phases and indeterminate form of CD underwent the examination, performed on a Cardios brand treadmill, with ERGO PC 13 software (Windows), Version 3.1. This system is capable of assessing clinical, hemodynamic, metabolic, electrocardiographic, and autonomic variables both during exercise and during recovery, allowing for an adequate diagnostic and prognostic definition of existing pathological conditions.

The Bruce protocol was used for the exercise stress test, which is a multi-stage protocol characterized by a progressive and predetermined increase in workload every 3 minutes. The exercise stress test allowed for the assessment of exercise-induced arrhythmias, the parasympathetic response during the recovery phase, and calculations of chronotropic competence, chronotropic reserve, and chronotropic index. These calculations are performed as follows: **a. Chronotropic Competence** – the ability of the heart rate to increase in proportion to physical exertion. An adequate response is considered an increase in heart rate greater than 80% of the chronotropic reserve; b. **Chronotropic Reserve** – the difference between the age-predicted maximal heart rate and the resting heart rate (CR: age-predicted maximal HR – resting HR); c. **Chronotropic Index (CI)** – one of the ways to demonstrate the inability of the heart rate to increase linearly with physical exertion. The index is the result of the ratio between the difference of the achieved heart rate and the resting heart rate, and the difference of the age-predicted maximal heart rate and the resting heart rate. A normal expected value is between 0.8 and 1.1. Values below 0.8 denote a response compatible with chronotropic incompetence and, consequently, an altered ANS response [45].

#### Heart rate variability interpretation.

Short-term HRV (5 minutes) was interpreted in the following ways: a. time domain; b. frequency domain. In Time Domain, we used linear statistical and geometrical methods. The linear statistical methods, such as SDNN and rMSSD indices were selected for evaluation. SDNN refers to overall ANS activity (sympathetic and parasympathetic) without distinction. rMSSD, whose analysis is based on adjacent RR intervals, refers to parasympathetic activity. The SD1 and SD2 indices were used in the geometric analysis (Poincaré plot). SD1 represents instantaneous, beat-to-beat HRV, reflecting parasympathetic influence. SD2 represents long-term HRV recordings, demonstrating sympathetic influence with vagal inhibition.

In frequency domain, we utilized the indices: a. high-frequency (hf) oscillatory component has a wave variation between 0.15 and 0.4 Hz, corresponds to respiratory modulation, an indicator of vagal nerve influence on the heart; b.

low-frequency (LF oscillatory component wave variation between 0.04 and 0.15 Hz, resulting from the joint action of vagal and sympathetic components on the heart, with sympathetic predominance; c. normalized LF/ normalized HF (LF/HF) ratio: reflect the sympathovagal balance on the heart. Normalized LF components and an increased LF/HF ratio were interpreted as sympathetic activity predominance. Normalized HF component and a reduced LF/HF ratio were interpreted as parasympathetic activity predominance.

In non-linear domain, the indices employed were: a. **DFA α1**: short-term detrended fluctuation analysis exponent estimates the self-similarity of HRV properties at short scales and is effective in clinical applications. It is capable of quantifying short-term changes caused by NN interval oscillations that can be affected by autonomic activation, reflecting the balance between sympathetic and parasympathetic branches, with predominance of sympathetic influence; b. **DFA α2**: long-term detrended fluctuation analysis exponent represented by the 1/f fraction, on a logarithmic scale, where f < 0.01 Hz. It contributes to understanding HRV, as fractal or self-similar measures provide a quantitative description of irregularities present in physiological signals. Unlike spectral techniques, fractal analysis studies the irregularity of the NN series assuming it is not characterized by any fixed-time scale. It is related to parasympathetic activity. A reduced DFA α2 indicates impairment of the parasympathetic branch or vagal regulation.

For the calculation of normalized components, the following formula was used: Absolute LF or HF component divided by the difference between total spectral power and the very low frequency (VLF) component multiplied by 100.

In long-term periods, HRV was assessed by dynamic electrocardiography (24-hour Holter monitoring) with evaluation of SDNN, SDANN, SDANNi, rMSSD, and pNN50 indices.

### Statistical analysis

Statistical analysis was performed with a description of numerical variables using medians, interquartile ranges, means, and standard deviations. Categorical variables were described in absolute values and percentages. Jamovi.org software was used for statistical analyses. The comparison of independent groups was performed for each variable following the procedure below: **a.** For independent sample: the non-parametric Mann-Whitney U Test was used, capable of determining whether observations from one population tend to be greater or smaller than another population.

## Results

### Clinical characteristics of the study population

The study population comprised 28 individuals, with 16 women (57.1%) and 12 men (42.9%). The mean age was 38.7 ± 16.6 years, with a body mass index of 27 kg/m², and a mean arterial pressure of 122/80 mmHg. These individuals originated from municipalities in the state of Amazonas in 89.3% of cases, and 10.7% were from Manaus and the capital’s peri-urban area. Infection was acquired via oral transmission in 85.7% of cases.

The electrocardiogram revealed the following findings: 1. compatible with normal patterns (60.7%), 2. diffuse ventricular repolarization abnormalities (21.4%), 3. left anterior fascicular block (7.1%), 4. right bundle branch block (7.1%), and 5. ventricular pre-excitation (3,5%). Echocardiogram results indicated normal ejection fraction and cavity diameters in all patients. During the acute phase, symptoms were non-specific, including fever, myalgia, and malaise. Epigastric pain was reported by one patient. No cardiological symptoms were reported by any patient.

The data are described in Table 1.

**Table 1:** Baseline characteristics of patients with acute Chagas disease treated with benznidazole.

Baseline Characteristics	G1 (n = 28)	G3 (n = 20)	*p-value*
**Age**	38,7 ± 16,6	39,9 ± 15,5	0.80
**BMI**	27 ± 2,6	27 ± 5,4	0.65
**SBP**	122 ± 22	123 ± 10	0.72
**DBP**	80 ± 13	78 ± 10	0.69
**Women**	16	11	0.78
**Men**	12	9	0,78
**Municipalities**			0.001*
**Manaus**	2	20	
**Lábrea**	7	0	
**Pau-Rosa**	1	0	
**Barreirinha**	11	0	
**Caraurari**	6	0	
**Tefé**	1	0	
**Transmission route**			
**Unknown**	3	–	
**Oral**	24	–	
**Vectorial**	1	–	
**EKG**			0.16
**Normal Patterns**	17	17	
**VRA**	6	0	
**LAFB**	2	0	
**RBBB**	1	1	
**RBBCD**	0	2	
**Ventricular pre-excitation**	1	0	
**Echocardiogram**			
**LVDD (mm)**	47,1 ± 3	46,4 ± 5	0.53
**LVEF %**	73 ± 6	72 ± 6	0.31

Values are expressed as mean (± SD); * p < 0,001 compared to groups G1 (acute disease) and G3 (control); **BMI**: Body Mass Index; **SBP**: Systolic Blood Pressure; **DBP**: Diastolic Blood Pressure; **EKG**: Electrocardiogram; **VRAs**: Ventricular Repolarization Abnormalities; **LAFB**: Left Anterior Fascicular Block; **RBBB**: Right Bundle Branch Block; **RBBCD**: Right Bundle Branch Conduction Delay; **LVDD**: Left Ventricular Diastolic Diameter; **LVEF**: Left Ventricular Ejection Fraction.

### Heart rate variability

Regarding heart rate variability, the results of analyses performed in the acute phase of CD, without treatment, for both long-term (Holter methodology) and short-term (5 minutes) periods are described in Table 2 and Table 3.

**Table 2:** Profile of 24-hour heart rate variability in the acute phase (untreated) versus control group.

24h HRV index	G1 n = 28	G3 n = 20	IC 95%	Effect Size (r_rb_)	*p-value*
**SDNN (ms)**	116 [72–186]	130 [84–197]	−33– − 4	0.27	0.11
**SDANN(ms)**	96 [54–177]	116 [72–189]	**−34– − 2**	**0.35**	**0.04**
**SDNNi (ms)**	46 [24–77]	56 [36–96]	**−14– − 3**	**0.29**	**0.04**
***r*MSSD (ms)**	25 [8–68]	34 [16–72]	−12–4	0.20	0.24
**pNN50 (ms)**	4 [0–40]	13 [8–57]	−6–3	0.14	0.40

#Rank-Biserial correlation.

Mann-Whitney U Test.

**Table 3:** Profile of short-term heart rate variability (5 minutes) in the acute phase (untreated) versus control group.

5-minute HRV index	G1 n = 28	G3 n = 20	IC 95%	Effect Size (r_rb_)	*p-value*
**Time Domain**					
**HR (bpm)**	77 [59–128]	68 [58–96]	2– − 18	0.47	0.01
**IRR (ms)**	781 [474–1028]	868 [622–982]	**−175– − 11.3**	**0.43**	**0.02**
***r*MSSD (ms)**	19.5 [5.4–68]	28 [10–79]	**−21.8– − 2.4**	**0.44**	**0.02**
**SDNN (ms)**	21.5 [5.2–51.6]	29 [13–76]	**−21– − 0.1**	**0.38**	**0.04**
**Frequency Domain**					
**HF (hertz)**	73 [6 –940]	228 [41–2951]	**−322– − 38**	**0.51**	**0.005**
**LF (hertz)**	60 [36.9–86]	44 [20–86]	**5–52**	**0.54**	**0.005**
**LF/HF**	1.5 [0.5–10.7]	0.8 [0.2–5.9]	**0.1–2.5**	**0.49**	**0.010**
**Non-linear Domain**					
**SD1**	14 [3.8–48]	25 [7–54]	**−17.4– − 3**	**0.54**	**0.005**
**SD2**	29.6 [5.5–59]	30 [13–78]	−16–7.7	0.16	0.39
**Alpha 1**	1.0 [0.6–1.6]	0.85 [0.5–1.4]	−0.1–0.4	0.27	0.15
**Alpha 2**	0.3 [0.2–1.1]	0.2 [0.1–0.7]	−0.09–0.20	0.21	0.28

#Rank-Biserial correlation.

Mann-Whitney U Test.

In the acute phase of Chagas Disease, alterations were observed in the 24-hour time-domain variables, specifically in SDANN (p-value = 0.04; r_rb_ = 0.35) and SDNN (p-value = 0.04; r_rb_ = 0,29). Such patterns suggest sympathovagal imbalance.

In the short-term period (5 minutes), the following altered indices were identified: heart rate (HR) with an increase in the acute phase group (p-value = 0.01; r_rb_ = 0.47); SDNN – reflecting a sympathovagal imbalance (p-value = 0.04; r_rb_ = 0.38); rMSSD – an index influenced by the parasympathetic autonomic nervous system (p-value = 0.02; r_rb_ = 0.44), all these alterations observed in the time domain.

In the frequency domain, alterations were observed in the following indices: High Frequency (HF) indicating an altered parasympathetic response (p-value = 0.005; r_rb_ = 0.51); Low Frequency (LF) suggesting an altered sympathetic response (p-value = 0.005; r_rb_ = 0.54); and LF/HF ratio demonstrating sympathovagal imbalance (p-value = 0.010; r_rb_ = 0.49). In the non-linear domain, the SD1 index, which indicates parasympathetic inhibition, showed a statistical difference compared to the control group (p-value = 0.005; r_rb_ = 0.54).

Table 4 and Table 5 show the results of heart rate variability for long-term and short-term periods, respectively, in the case group after trypanocidal treatment (benznidazole).

**Table 4:** Profile of 24-hour heart rate variability in the post-treatment phase versus control group.

24-hour HRV index	G2 n = 28	G3 n = 20	IC 95%	Effect Size^#^ (r_rb_)	*p-value*
**SDNN (ms)**	131 [88–200]	130 [84–197]	−15–16	0.02	0.90
**SDANN (ms)**	113 [78–164]	116 [72–189]	−20–10	0.17	0.53
**SDNNi (ms)**	55 [38–105]	56 [36–96]	−20–10	0.29	0.53
***r*MSSD (ms)**	38 [5–87]	34 [16–72]	−7–14	0.10	0.55
**pNN50 (ms)**	13.5 [0–31]	13 [8–57]	−4–9	0.10	0.54

#Rank-biserial correlation.

Mann-Whitney U Test.

**Table 5:** Profile of short-term heart rate variability in the post-treatment phase versus control group.

5-minute HRV index	G2 follow-up n = 17	G3 n = 20	IC 95%	Effect Size^#^ (r_rb_)	*p-value*
**Time Domain**					
**HR (bpm)**	69 [49–110]	68 [58–96]	−3.4–8	0.17	0.38
**IRR (ms)**	859 [369–1222]	868 [622–982]	−79–44	0.14	0.47
***r*MSSD (ms)**	24 [6.7–53.5]	28 [10–79]	−14–4	0.20	0.29
**SDNN (ms)**	25.3 [10–52]	29 [13–76]	−16–1.5	0.31	0.13
**Frequency Domain**					
**HF (hertz)**	239 [29–1276]	228 [41–2951]	−241–48	0.01	0.24
**LF (hertz)**	54.6 [23.7–91.5]	44 [20–86]	−24–3.8	0.22	0.21
**LF/HF**	1.2 [0.3–10.7]	0.8 [0.2–5.9]	−0.2 −1.0	0.24	0.20
**Non-linear Domain**					
**SD1**	17 [4.7–38]	25 [7–54]	−17.4– − 3	0.54	0.08
**SD2**	31 [12–63]	30 [13–78]	−12–8.7	0.33	0.83
**Alpha 1**	0.9 [0.5–1.5]	0.85 [0.5–1.4]	−0.1–0.03	0.15	0.43
**Alpha 2**	0.4 [0.2–1.2]	0.2 [0.1–0.7]	−0.2–0.1	0.24	0.21

#Rank-biserial correlation.

Mann-Whitney U Test.

In the post-treatment phase, all measured parameters exhibited sustained normalization, indicating a restoration of autonomic nervous system function.

The 5-minute HRV analysis showed no statistically significant changes in the indices.

Table 6 and Table 7 describe the intragroup comparisons between pre- and post-trypanocidal treatment phases.

**Table 6:** Profile of 24-hour heart rate variability in the Chagas group during pre- and post-treatment periods.

24h HRV Index	G1 n = 28	G2 follow-up n = 28	IC 95%	Effect Size^#^ (r_rb_)	*p-value*
**SDNN (ms)**	116 [72–186]	131 [88–200]	**−24–4**	**0.50**	**0,02**
**SDANN (ms)**	96 [54–177]	113 [78–164]	−34– − 2	0.35	0,12
**SDNNi (ms)**	46 [24–77]	55 [38–105]	**−19– − 5**	**0.68**	**0,002**
**rMSSD (ms)**	25 [8–68]	38 [5–87]	−16– − 0.01	0.42	0,06
**pNN50 (ms)**	4 [0–40]	13.5 [0–31]	−6–1.5	0.38	0,14

#Rank- biserial correlation.

Mann-Whitney U Test.

**Table 7:** Profile of short-term heart rate variability (5 minutes) in the Chagas group pre- and post-treatment.

5-minutes HRV index	G1 n = 28	G2 follow-up n = 17	IC 95%	Effect Size^#^ (r_rb_)	*p-value*
**Time Domain**					
**HR (bpm)**	77 [59–128]	69 [49–110]	**0.3–19**	**0.57**	**0.04**
**IRR (ms)**	781 [474–1028]	859 [369–1222]	**−173– − 0.02**	**0.52**	**0.04**
**rMSSD (ms)**	19.5 [5.4–68]	24 [6.7–53.5]	−10.8–0.45	0.21	0.06
**SDNN (ms)**	21.5 [5.2–51.6]	25.3 [10–52]	3.8– − 9.6	0.23	0.45
**Frequency Domain**					
**HF (hertz)**	73 [6 –940]	239 [29–1276]	−299–127	0.19	0.12
**LF (hertz)**	60 [36.9–86]	54.6 [23.7–91.5]	−21–6.3	0.25	0.30
**LF/HF**	1.5 [0.5–10.7]	1.2 [0.3–10.7]	−0.5–1.9	0.29	0.50
**Non-linear Domain**					
**SD1**	14 [3.8–48]	17 [4.7–38]	**−7.9–0.15**	**0.54**	**0.04**
**SD2**	29.6 [5.5–59]	31 [12–63]	−13.6–6.7	0.20	0.48
**Alpha 1**	1.0 [0.6–1.6]	0.9 [0.5–1.5]	−0.2–0.4	0.12	0.67
**Alpha 2**	0.3 [0.2–1.1]	0.4 [0.2–1.2]	**0.2–0.01**	**0.65**	**0.03**

#Rank-biserial correlation.

Mann-Whitney U Test.

In the intragroup comparison, during the long-term period (24 h), the indices that demonstrated statistically significant alterations were: SDNN (p = 0.02; r_rb_ = 0.50) and SDNNi (p = 0.002; r_rb_ = 0.68), indicating the maintenance of sympathovagal imbalance.

In the intragroup comparison, during the short-term period (5 minutes), the indices that demonstrated statistically significant alterations were: heart rate (HR) (p = 0.04; r_rb_ = 0.57), SD1 (p = 0.04; r_rb_ = 0.54) and Alpha 2 (p = 0.03; r_rb_ = 0.65) and these indices are associated with inhibition of the parasympathetic response.

The Table 8 shows the autonomic response observed in the exercise stress test performed in the post-treatment phase.

**Table 8:** Autonomic profile observed in the exercise stress test in the post-treatment phase.

Index	G2 follow-up (n = 13)	G3 (n = 20)	IC 95%	Effect Size^#^ (r_rb_)	p-value*
**HR max**	159 [134–184]	152 [134–170]	23.7–8.5	0.34	0.34
**CR**	77 [53–101]	80 [55–105]	15.4– − 20	0.10	0.34
**CI**	0.76 [0.56–0.96]	0.76 [0.59–0.93]	0.13– − 0.14	0.001	0.97
**HRR**	142 [128–156]	124 [105–143]	**31–5.6**	**1.05**	**0.006**

* Student’s t-test; HR: heart rate; CR: chronotropic reserve; CI: chronotropic index; HRR: heart rate recovery.

Heart Rate Recovery (HRR) was observed to show an altered response and statistical significance (p = 0.006; d = 1.05). This index is associated with an inhibition of the parasympathetic response.

## Discussion

Autonomic nervous system dysfunction is involved in arrhythmogenic mechanisms and associated with sudden death in Chronic Chagasic Cardiomyopathy (CCC) [1,2]. One of the prominent prognostic biomarkers is Heart Rate Variability (HRV) analysis, which reflects beat-to-beat changes in RR intervals, related to the continuous interaction between the two branches of the autonomic nervous system. These branches are the parasympathetic and sympathetic systems. In other words, HRV indicates the body’s ability to adjust heart rate in response to internal and external stimuli, demonstrating the balance between sympathetic and parasympathetic activity [2,32–34,53,54].

The parasympathetic system is responsible for greater heart rate or RR interval variability, while the sympathetic system produces lower heart rate variability. A reduction in HRV is associated with a higher risk of cardiovascular mortality. Since the beginning of studies on Chagas disease, the presence of a mononuclear infiltrate with ganglionic lesion, neuronal reduction, and a numerical decrease in parasympathetic nerve cells has been described, characterizing a parasympathetically denervated cardiomyopathy with sympathetic predominance [32,33,37,53,55].

For HRV assessment, with the aim of investigating autonomic dysfunction, oscillations in consecutive heart beat intervals (RR intervals) are studied. These are related to the influence of the ANS on the sinus node over a specific period of time (2, 5, 15 minutes up to 24 hours). HRV analyses are performed using linear methods, in the time and frequency domains, and non-linear methods. This allows for the evaluation of characteristic responses of the parasympathetic and sympathetic branches, as well as sympathovagal imbalance [2,15].

This study, for the first time, characterized the autonomic nervous system in patients from the Western Amazon region with ACD using heart rate variability, revealing the following acute phase findings: (a) long-term indices demonstrated sympathovagal imbalance; and (b) short-term indices showed responses characterized by sympathovagal imbalance and parasympathetic inhibition. In the post-treatment follow-up phase, all measured parameters exhibited sustained normalization, indicating a restoration of autonomic nervous system function. Although heart rate variability assessment indices were normalized, a parasympathetic inhibition response was observed in the recovery phase of the exercise stress test.

Regarding the demographic characteristics of the studied population, it was observed that most individuals with Acute Chagas Disease (ACD) came from other localities distant from the capital, affecting both sexes with no predominance between them, and the most common route of contamination was oral. This trend was consistent with recent findings published concerning Amazonas state, where the diagnosis of acute disease was linked to outbreaks and micro-outbreaks among individuals from other municipalities and associated with the consumption of palm fruits such as açaí and patauá [9,10,13,16–20,56].

In terms of clinical manifestations, the presence of non-specific symptoms such as fever, adenomegaly, malaise, and the absence of associated cardiac manifestations was noted, meaning the symptoms were milder and evolved benignly [23,57]. Pinto et al. (2013) reported similar behavior in a cohort studied in another Amazonian region [108]. Such behavior may be related to genetic resistance to Chagas Disease (CD) and the immunological response to the parasite. These genetic mutations, related to the PPP3CA gene, occurred thousands of years ago in indigenous peoples and were passed down to their descendants, ensuring the perpetuation of a more resistant population group [58].

Cardiologic evaluation demonstrated that the electrocardiogram in the acute phase was normal in most cases. However, the second most common morphology was diffuse repolarization abnormality. This pattern has been demonstrated in other studies and can be explained by the inflammatory process or residual myocardial fibrosis in infected individuals [27]. In the Bambuí cohort study, it was evidenced that the persistence of electrocardiographic changes would be associated with progression to CCDC over 30 years of population follow-up [59]. The echocardiogram was normal in all cases. This result indicates a benign aspect of the infection in the region. Pinto et al. (2013) found similar results in another study in the Amazonian region [57].

The autonomic nervous system response was altered in both the long-term (24h) and short-term (5 min) periods, in the acute phase. The main modifications in the acute phase were sympathovagal imbalance and parasympathetic branch inhibition, regardless of normal electrocardiogram and echocardiogram results. Several studies have demonstrated that autonomic dysfunction precedes the development of ventricular dysfunction and heart failure in patients with indeterminate and digestive forms of Chagas disease [31,38,60,61]. Intragroup comparisons revealed that the acute disease group exhibited greater autonomic nervous system dysfunction than the etiologically treated group, a finding consistent with the expected response when specific treatment is administered. This autonomic dysfunction response finding in acute phase can be explained by three mechanisms: direct parasitism of neurons, degeneration caused by periganglionic inflammation, and antineuronal autoimmune reaction with the presence of autoantibodies against muscarinic and beta-adrenergic receptors [14,15,27,42,62,63].

The sympathovagal imbalance and parasympathetic response inhibition observed in the short-term, characterized by altered responses in rMSSD, HF (linear domain), and SD1 (non-linear domain) indices found in our analysis, are consistent with the first studies on autonomic dysfunction in Chagas disease conducted by Köberle, who proved the presence of parasympathetic neuronal destruction and depopulation, characterizing a parasympathetically denervated cardiomyopathy with sympathetic predominance [33,34,53]. In addition to this behavior in the acute phase, the presence of sympathovagal imbalance, justified by sympathetic dysautonomia, was also observed in previous studies and justified by direct destruction and the presence of antimuscarinic and anti-beta2 adrenergic autoantibodies [14,15,42,61–66].

Another mechanism pointed out as responsible for sympathovagal imbalance is the cholinergic inflammatory pathway, where the inflammatory process and the autonomic nervous system reciprocally regulate each other, determining a pro- or anti-inflammatory response. In our study, more altered HRV indices were evidenced in the acute phase of the disease, with partial normalization after 6 months. Llaguno et al. (2011) demonstrated that different titrations of certain cytokines would determine greater inflammatory infiltrate and would be associated with greater autonomic dysfunction [42]. Machado et al. (2012) proposed that cardiac autonomic denervation would have a reduced capacity to inhibit the pro-inflammatory response of Th1 cells, contributing to a greater magnitude of these cells’ response. This would contribute to an increase in the cardiac inflammatory process, with greater destruction of autonomic fibers and neurons, perpetuating the positive feedback process in CCDC and demonstrating the role of the autonomic nervous system in modulating the immune response to T. cruzi [55].

Corroborating the 5-minute HRV findings, the exercise stress test response demonstrated that heart rate recovery was compatible with a reduction in the inhibitory response of the parasympathetic branch. The results we present in this study indicate an altered autonomic nervous system response, characterized by sympathovagal imbalance and a reduction in the inhibitory action of the parasympathetic branch, both in the pre-treatment acute phase and 6–12 months post-specific treatment.

Although the normalization of heart rate variability parameters could serve as an additional justification for the favorable clinical course in individuals treated for Acute Chagas Disease (ACD) at FMT-HVD, the autonomic nervous system dysfunction observed during the acute phase still highlights the need for longer-term follow-up of this population to assess the long-term progression and outcomes of Chagas disease in our region.

Patient follow-up in Chagas disease is inherently challenging due to the disease’s low prevalence and the remote, hard-to-access Amazonian regions from which patients originate. High transportation costs, coupled with prolonged river travel, further impede consistent patient attendance. Moreover, local health facilities in these municipalities of origin often possess restricted capabilities for adequate follow-up. Consequently, these multifaceted logistical and infrastructural obstacles significantly compromise adherence to recommended long-term follow-up protocols. These unique challenges collectively justify the study’s limited patient cohort and its extended follow-up duration.

The analysis of heart rate variability has as its main limitations the capture of a specific moment of autonomic activity, the influence of external environmental factors, and the difficulty in demonstrating circadian changes and long-term trends. To mitigate these limitations, we opted for short- and long-term analysis, in addition to following all recommended protocols for the use of this biomarker, both during biomarker collection and statistical analysis. Thus, our study demonstrates a trend in the autonomic nervous system response, opening perspectives for further studies in the population with Chagas disease.

## Conclusions

This study demonstrated a tendency towards sympathovagal imbalance and a reduced inhibitory response of the parasympathetic branch in the acute phase. While normalization of these findings was observed after benznidazole treatment, analysis of the exercise stress test revealed that the pattern of reduced parasympathetic inhibition persisted 8 months post-treatment in individuals diagnosed with Acute Chagas Disease (ACD) treated at FMT-HVD.
